# Discovery of *GJC1* as a prognostic biomarker in glioma cells: insights into its cell-cycle relationship and differential expression in non-neuronal cells

**DOI:** 10.3389/fncel.2024.1440409

**Published:** 2024-09-18

**Authors:** Xiangtian Ji, Xin Chen, Guozhong Lin, Kaiming Ma, Junhua Yang, Xiaofang Zhao, Suhua Chen, Jun Yang

**Affiliations:** ^1^Department of Neurosurgery, Peking University Third Hospital, Beijing, China; ^2^Center for Precision Neurosurgery and Oncology of Peking University Health Science Center, Beijing, China

**Keywords:** glioma, non-neuronal cells, single-cell RNA sequencing, bioinformatics, cell cycle regulation, oncology drugs

## Abstract

**Background:**

Gliomas, originating from the most common non-neuronal cells in the brain (glial cells), are the most common brain tumors and are associated with high mortality and poor prognosis. Glioma cells exhibit a tendency to disrupt normal cell-cycle regulation, leading to abnormal proliferation and malignant growth. This study investigated the predictive potential of *GJC1* in gliomas and explored its relationship with the cell cycle.

**Methods:**

Retrospective analysis of RNA-seq and single-cell sequencing data was conducted using the Chinese Glioma Genome Atlas (CGGA) and The Cancer Genome Atlas (TCGA) databases. The differential expression of *GJC1* in gliomas with various pathological features and in different non-neuronal cell groups was analyzed. Functional data were examined using gene set variation analysis (GSVA). Furthermore, CellMiner was used to evaluate the relationship between *GJC1* expression and predicted treatment response across these databases.

**Results:**

*GJC1* expression was enriched in high-grade gliomas and 1p/19q non-codeletion gliomas. *GJC1* enrichment was observed in classical and mesenchymal subtypes within the TCGA glioma subtype group. In single-cell subgroup analysis, *GJC1* expression was higher in glioma tissues compared to other non-neuronal cells. Additionally, the TCGA classical subtype of glioma cells exhibited more *GJC1* expression than the other subgroups. *GJC1* emerged as an independent prognostic factor for overall survival in glioma. GSVA unveiled potential mechanisms by which *GJC1* may impact cell-cycle regulation in glioma. Finally, a significant correlation was observed between *GJC1* expression and the sensitivity of multiple anti-cancer drugs.

**Conclusion:**

These findings confirmed *GJC1* as a novel biomarker and provided insights into the differential gene expression in non-neuronal cells and the impact of the cell cycle on gliomas. Consequently, *GJC1* may be used to predict glioma prognosis and has potential therapeutic value.

## 1 Introduction

Non-neuronal cells, primarily glial cells, play crucial roles in the nervous system by providing support, protection, and nourishment to neurons. These cells, including glial cells, fibroblasts, and other supporting cells, retain the ability to divide, making them more susceptible to mutations and malignant transformation. Consequently, nearly all malignant tumors in the central nervous system originate from non-neuronal cells (Molinaro et al., 2019). Gliomas are the most prevalent primary malignant brain tumors in adults arising from glial cells of the central nervous system. Gliomas account for over 80% of malignant brain tumors, with an incidence rate of approximately 6 per 100,000 individuals. Gliomas are classified by grade according to the World Health Organization (WHO) Central Nervous System Tumor Classification based on pathology. WHO grade I gliomas pose low risk and can often be surgically removed. Low-grade WHO grade II gliomas are well-differentiated and exhibit benign traits but tend to recur and progress. High-grade WHO grade III-IV gliomas are anaplastic, malignant, and have a poor prognosis (Alexander and Cloughesy, 2017). Recent therapeutic advances have led to the development of targeted inhibitors, immunotherapies, and novel delivery systems. Small molecule inhibitors of mutant IDH1/2 enzymes have shown promising results in IDH-mutant gliomas. PARP inhibitors exploit homologous recombination deficiency in IDH-wildtype gliomas (Mellinghoff et al., 2023). Inhibitors targeting receptor tyrosine kinases such as EGFR, PDGFR, and c-Met are being tested clinically (Eskilsson et al., 2018). Immunotherapies being evaluated include checkpoint inhibitors and chimeric antigen receptor T-cell therapy (Majzner et al., 2022). Convection-enhanced delivery has shown improved distribution of chemotherapeutics in the brain. Despite these advancements, the prognosis remains poor, and resistance inevitably emerges.

The relationship between non-neuronal cells and gliomas is crucial. Various non-neuronal cells, including T cells, macrophages, and astrocytes, play significant roles in tumor development and progression. Gliomas interact with these cells to create an immunosuppressive environment. Understanding the relationship between genomic alterations in non-neuronal cells and cell cycle dysregulation can reveal new therapeutic vulnerabilities for brain tumors. The transformation of glial cells into glioma cells often involves the disruption of key cell-cycle regulators such as p53, Rb, and INK4a/ARF. The loss of these tumor suppressors leads to abnormal proliferation. Identifying druggable targets could result from elucidating subtype-specific dependencies on cyclins, cyclin-dependent kinases (CDKs), and checkpoints (Zhao et al., 2023). Glioblastoma can be classified into four distinct subtypes based on its molecular characteristics: pro-neurogenic, neurogenic, classic, and mesenchymal (Wang et al., 2017). Neftel et al. (2019) demonstrated four discrete cellular subtypes within glioblastoma at the single-cell level. Based on the similarity of these subtypes to non-neuronal cell groups, these subtypes include neural progenitor-like, oligodendrocyte progenitor-like, astrocyte-like, and mesenchymal-like states, and they can be associated with their corresponding ontological subtypes. Integrating molecular classification with cell cycle profiles and therapeutic response patterns will advance precision therapy for patients with glioma. Achieving a thorough comprehension of the pivotal molecules and mechanisms implicated in gliomas is crucial for their diagnosis and treatment. *GJC1*, situated on human chromosome 17, encodes the gap junction gamma-1 protein (connexin 45, or Cx45). Connexins are a group of protein molecules that form gap junctions (GJs) for intercellular communication. Cx45, a major component of GJs (Choi et al., 2020), showed decreased expression in colorectal cancer owing to *GJC1* promoter hypermethylation (Sirnes et al., 2011). Additionally, research has suggested that Cx45 plays a tumor-suppressive role in melanoma cells (Saito et al., 2023). However, the functional role of *GJC1* in gliomas remains unexplored, and further investigation is warranted.

In this study, we performed a comprehensive analysis of *GJC1* expression profiles across various glioma subtypes using data from the Chinese Glioma Genome Atlas (CGGA) and The Cancer Genome Atlas (TCGA) were used. Our study aimed to clarify potential correlations between *GJC1* expression levels, clinicopathological characteristics, and overall survival (OS) among patients with glioma. Additionally, we scrutinized *GJC1* expression at the single-cell level across distinct glioma subgroups and different non-neuronal cells. These findings illuminate the significance of *GJC1* in gliomas and provide valuable insights for future therapeutic strategies.

## 2 Methods

### 2.1 Glioma transcriptome sequencing database

Standardized RNA-seq and clinical data encompassing details on 693 patients with glioma were obtained from the CGGA website (http://www.cgga.org.cn/; Zhao et al., 2021) to investigate gene expression profiles and clinical details. The primary objective of this analysis was to assess OS outcomes. A comprehensive analysis of the standardized RNA-seq data and *GJC1* gene expression was conducted using R 3.3.1. Additionally, datasets from TCGA (http://tcga-data.nci.nih.gov) were used to assess the differential expression patterns of *GJC1* across various glioma grades.

### 2.2 Gene set variation analysis

The list of genes associated with the cell cycle was obtained from the AmiGO (RRID:SCR_002143) 2 portal (http://amigo.geneontology.org/amigo). Functional enrichment scores for each glioblastoma multiforme sample were calculated using the default parameters of the “gsva” package in R. A heatmap was generated using the pheatmap (RRD:SCR_016418) package within R to visualize the enrichment results (Hänzelmann et al., 2013). Pearson correlation analysis was employed to evaluate the correlation between *GJC1* and the cell cycle.

### 2.3 Functional enrichment analysis

The most pertinent genes associated with *GJC1* or a characteristic gene list from the cell cluster were submitted to the Database for Annotation, Visualization, and Integrated Discovery (DAVID RRID:SCR_00188, v6.8). Official gene symbols were used as identifiers, and *Homo sapiens* was specified as the species. Subsequently, Gene Ontology (GO) and Kyoto Encyclopedia of Genes and Genomes (KEGG) pathway analyses were conducted to identify enrichment results. This study presented the top five results, and they were organized in ascending order of *p*-value (*p* < 0.05).

### 2.4 Single-cell sequencing database

The single-cell RNA-sequencing (scRNA-seq) database, which includes comprehensive clinical and follow-up information for all patients in this study, is available on the CGGA website (http://www.cgga.org.cn). Single cells were sequenced using a HiSeq 4,000 (Illumina, San Diego, CA). For coculture modes, scRNA-seq libraries were prepared following the SMART-Seq2 Genomics protocol as previously outlined. Subsequently, these libraries were sequenced on a HiSeq 2000 (Illumina) to generate 150-bp paired-end reads.

### 2.5 Cell clustering

The “seurat” package (version 4.0; Patel et al., 2014) was used to conduct cell clustering in patients with glioblastoma. The standard preprocessing workflow for scRNA-seq results, as outlined in the literature, was followed. Molecular markers for each cluster of different cells were obtained from the CellMarker website (http://xteam.xbio.top/CellMarker). In this study, cell clusters with high *CD3E* and *CD3D* expression, high *CD163* and *CD68* expression, high *FA2H* and *MBP* expression, and high *PTPRZ1* and *BCAN* expression were defined as T-lymphocytes, tumor-associated macrophages, oligodendrocytes, and glioma tumor cells, respectively.

### 2.6 Statistical analysis

All statistical data and figures were analyzed using SPSS (version 22.0; IBM, Armonk, NY), R 3.3.1 (R Foundation, Vienna, Austria), and GraphPad Prism 5.0 (GraphPad Software, San Diego, CA). Kaplan–Meier curves were constructed, and log-rank tests were conducted to assess the survival predictive performance of *GJC1* and the risk score. Univariate and multivariate Cox regression analyses were performed to examine the relationship between variables and OS. The nomogram was generated using SPSS, and prognostic ability was assessed using the “survival” and “survminer” packages in R. The receiver operating characteristic curves were plotted using the “survivalROC” package in R. Statistical significance was defined as *p ≤* 0.05.

### 2.7 *GJC1* and drug response

The relationship between *GJC1* expression and drug responsiveness was established using CellMiner (http://discover.nci.nih.gov/cellminer/). CellMiner is a specialized query tool and database designed to assist cancer researchers in integrating and evaluating molecular and pharmacologic data for the NCI-60 tumor cell lines (Shankavaram et al., 2009; Reinhold et al., 2012). The NCI-60 comprises 60 unique human tumor cell lines and is utilized by the National Cancer Institute Developmental Therapeutics Program for screening over 100,000 chemical compounds and natural products.

## 3 Results

### 3.1 *GJC1* expression levels were significantly enriched in glioma samples exhibiting malignant molecular markers and have a poor prognostic effect

**Figure 1:**
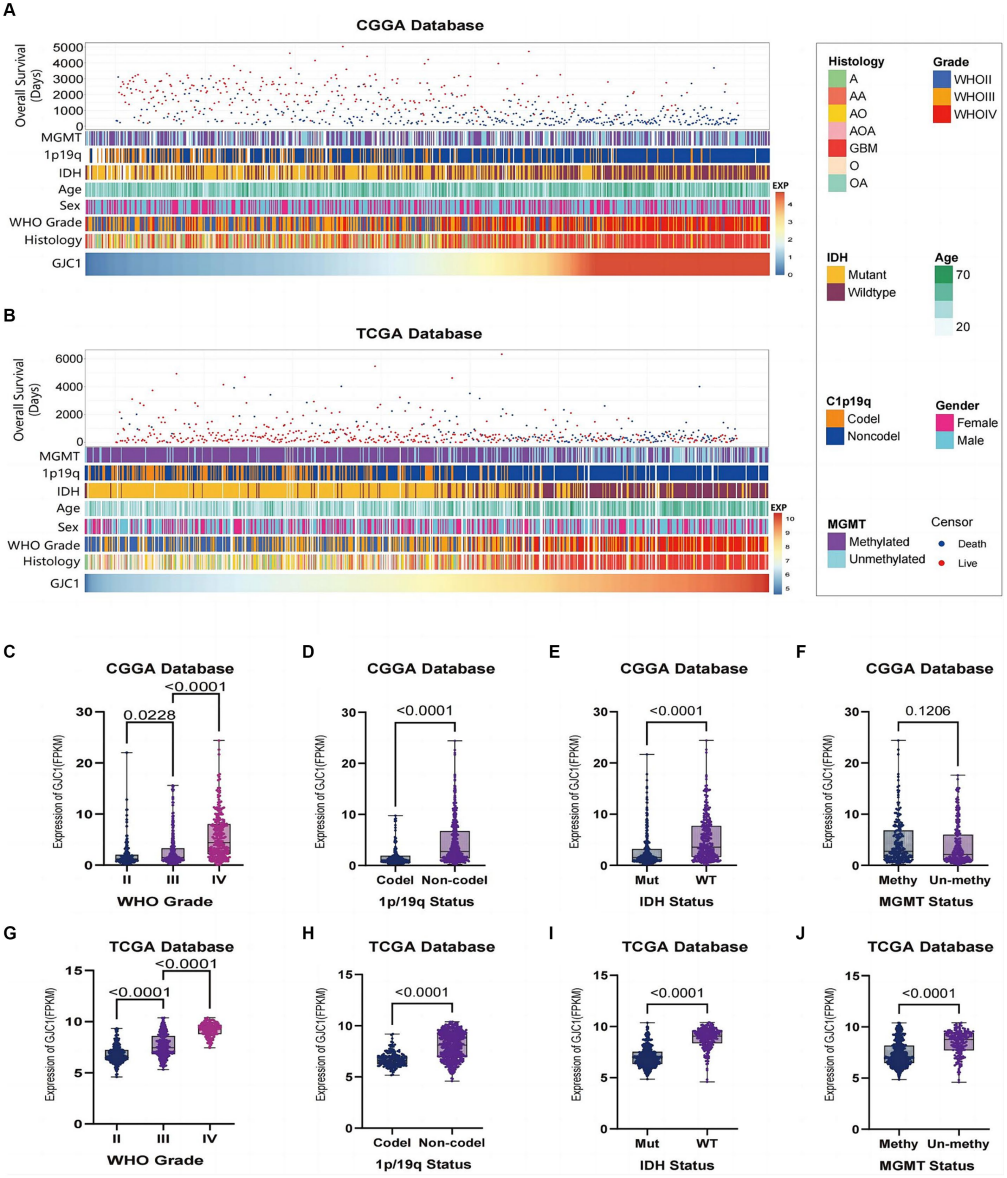
Relationship between *GJC1* and clinicopathological characteristics of gliomas. **(A)** Overview of *GJC1*-related clinicopathological features of gliomas in the CGGA database. **(B)** Overview of *GJC1*-related clinicopathological features of gliomas in the TCGA database. **(C,G)** *GJC1* expression showed a significant increase (one-way analysis of variance) in higher-grade gliomas in the CGGA and TCGA databases. **(D,H)** *GJC1* expression exhibited a significant increase (unpaired *t*-test) in gliomas without isocitrate dehydrogenase (*IDH*) mutation in the CGGA and TCGA databases. **(E,I)** *GJC1* expression demonstrated a significant increase (unpaired *t*-test) in gliomas without 1p/19q codeletion in the CGGA and TCGA databases. **(F,J)** *GJC1* expression was elevated in *MGMT* promoter–unmethylated gliomas. This distinction reached statistical significance (unpaired t-test) in the TCGA database but not in the CGGA database. CGGA, Chinese Glioma Genome Atlas; TCGA, the Cancer Genome Atlas.

In this study, we observed distinct clinical and pathological characteristics among patients with glioma with different levels of *GJC1* expression. A significant asymmetric distribution was observed in the malignant progression of O6-methylguanine-DNA methyltransferase (*MGMT*) promoter methylation status, 1p/19q co-deletion status, *IDH* mutation status, glioma grading, and histological types of samples with elevated expression levels of *GJC1* in both the CGGA and TCGA datasets (Figures 1A,B). We then conducted Kaplan–Meier analyses based on the CGGA and TCGA databases to explore the prognostic prediction value of *GJC1* in patients with glioma. In the CGGA database, patients with higher *GJC1* expression levels exhibited significantly shorter OS (median survival: 518 days) than those with lower *GJC1* expression (median survival: 1559 days) (Supplementary Figure S1A). Furthermore, the prognostic value of *GJC1* was verified in the TCGA database (Supplementary Figure S1B). Additionally, utilizing data from TCGA, we comprehensively assessed the sensitivity and specificity of *GJC1* at different time points through receiver operating characteristic curve analysis. The area under the curve for *GJC1* in the glioma cohort consistently exceeded 68% (Supplementary Figures S1C,D).

Glioma samples from the CGGA and TCGA datasets were categorized according to the WHO 2021 classification of gliomas, and *GJC1* expression was compared between each group. The findings indicated that *GJC1* expression levels escalated with high pathological grades (Figures 1C,G). Additionally, a notable enrichment of *GJC1* expression was observed in glioma samples with wild-type *IDH* and 1p/19q non-codeletion (Figures 1D,E,H,I). In the TCGA datasets, *GJC1* expression exhibited significant enrichment in samples with no *MGMT* promoter methylation (Figure 1J), whereas in the CGGA datasets, this difference was not statistically significant (Figure 1F). These results suggest that *GJC1* could serve as a potential biomarker for the diagnosis of gliomas.

### 3.2 *GJC1* Expression levels exhibit a preference for specific subtypes in glioblastoma

**Figure 2:**
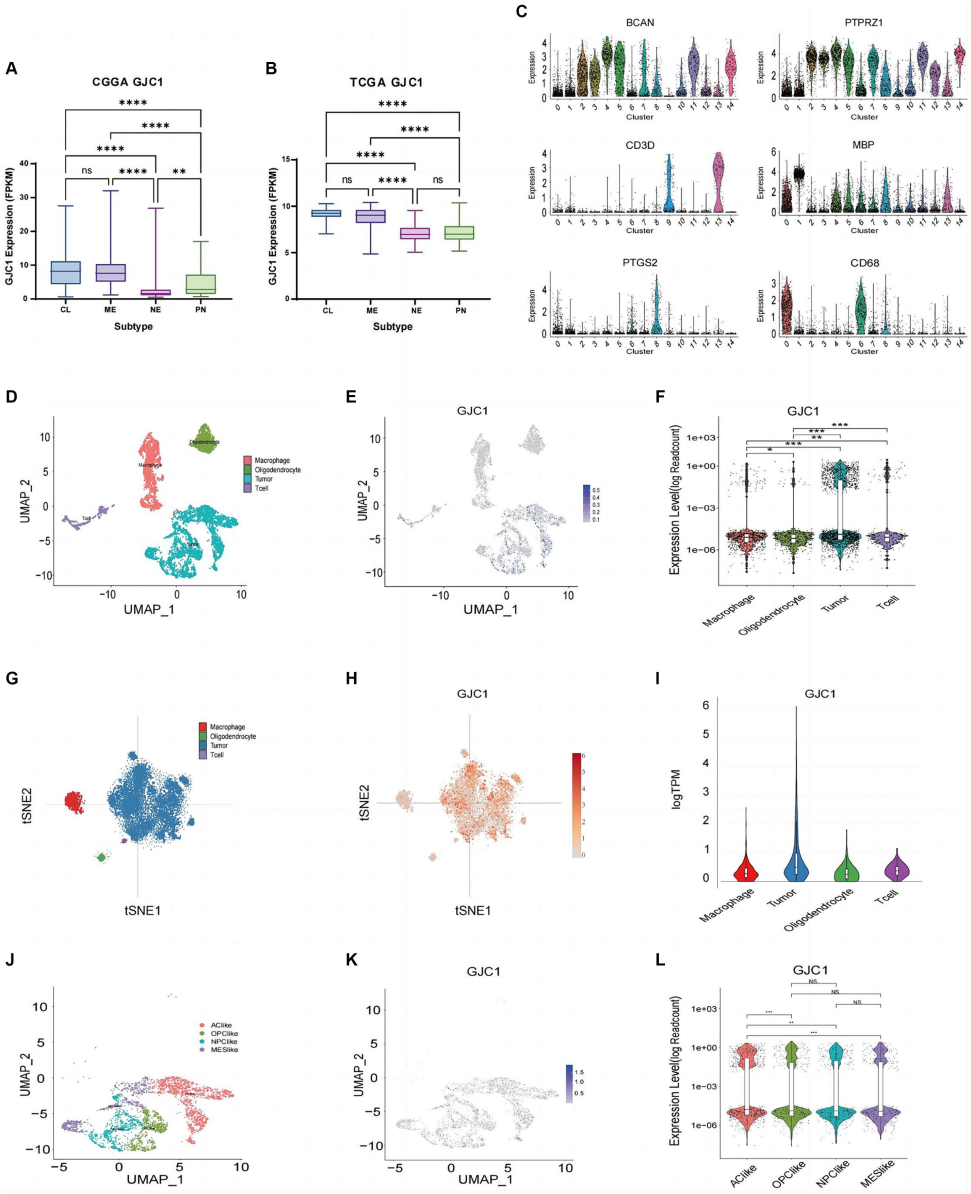
*GJC1* expression in various glioma subtypes. **(A,B)** *GJC1* expression levels across different molecular subtypes were evaluated using bulk tumor sequencing data from CGGA_325 and TCGA datasets. **(C)** Single-cell RNA (scRNA) sequencing data from the CGGA database were categorized into four clusters based on the molecular markers in figure **(C)**. Clusters of cells with high expression of CD3E and CD3D were defined as T-lymphocytes, clusters of cells with high expression of CD163 and CD68 were defined as macrophages, clusters of cells with high expression of FA2H and MBP were defined as oligodendrocytes, and clusters of cells with high expression of PTPRZ1 and BCAN were glioma tumor cells. **(D)** Four cell clusters presented in UMAP. **(E)** Blue scatter plots depict the distribution of *GJC1* expression in four clusters. **(F)** Violin plots illustrating the expression levels of *GJC1* in the four cell clusters. **(G)** Cell subtypes from isocitrate dehydrogenase (*IDH*)-wild-type glioblastomas in a public database (GSE131928). **(H)** Red scatter plots illustrate the distribution of *GJC1* expression in four clusters in a public database (GSE131928). **(I)** *GJC1* expression in various cells from *IDH*-wildtype glioblastomas in a public database (GSE131928). **(J)** Tumor cells were isolated and classified into four distinct cell clusters based on the scRNA expression level. **(K)** *GJC1* expression is depicted in blue scatter spots. **(L)** *GJC1* was predominantly expressed in AC-like subtypes compared to the other three subtypes. **p < 0.01, ***p < 0.001; NS, no statistical significance; CGGA, Chinese Glioma Genome Atlas; TCGA, The Cancer Genome Atlas; AC, astrocyte-like cell; OPC, oligodendrocyte progenitor cell; NPC, neural progenitor cell; MES, mesenchymal.

In the clinical diagnosis and treatment of glioblastoma, molecular subtypes provide valuable insights for predicting patient outcomes (Neftel et al., 2019). Based on extensive expression data studies in TCGA, the most common classification for glioblastoma is the Glioma Transcriptome Subtype. Within this classification, glioblastoma can be divided into four subtypes: classical (TCGA-CL), mesenchymal (TCGA-ME), proneural (TCGA-PN), and neural (TCGA-NE). Among these, the proneural (PN) subtype (Wang et al., 2017) typically exhibits a comparatively better prognosis, whereas the classical and mesenchymal subtypes tend to be more invasive and are associated with a poorer prognosis. In this study, *GJC1* expression was investigated in low-grade gliomas and glioblastoma samples from the TCGA and CGGA datasets. Elevated *GJC1* expression was associated with the NE and PN subtypes in the CGGA and TCGA datasets (Figures 2A,B). This further emphasizes the correlation between high *GJC1* expression and unfavorable prognosis. In gliomas, various non-neuronal cells play crucial roles. For instance, macrophages often support tumor growth, typically exhibiting an M2-like phenotype that is anti-inflammatory and promotes tissue repair, aiding in tumor immune evasion. They release growth factors and cytokines that enhance tumor cell survival and proliferation, thereby supporting tumor growth. To determine which non-neuronal cell type GJC1 might be regulating in gliomas, we further analyzed GJC1 expression from a single-cell perspective to elucidate its role in gliomas. We downloaded the single-cell sequencing database from the CGGA (DataSet ID: scRNA-seq, Platform: STRT-seq). In the single-cell sequencing data sourced from the CGGA database, cells were categorized into 15 clusters using *CD3D*, *MBP*, *CD68*, *PTGS2*, *PTPRZ1*, and *BCAN*. Clusters 9 and 13 were classified as T cells based on *CD3D* expression; cluster 1 was classified as oligodendrocytes based on *MBP*; clusters 0, 6, and 8 were classified as macrophages based on *CD68* and *PTGS2*; and clusters 2–5, 7, 10–12, and 14 were classified as tumor cells based on *PTPRZ1* and *BCAN* (Figures 2C,D). Upon defining each cluster, the expression pattern of GJC1 was observed across various non-neuronal cell types, including T cells, oligodendrocytes, macrophages, and tumor cells, and its presence was revealed across this diverse spectrum of non-neuronal cell populations (Figures 2E,F); notably, tumor cell clusters exhibited higher levels of *GJC1* expression. Additionally, through the analysis of public scRNA-seq datasets (GSE131928), we further confirmed that elevated *GJC1* expression was predominantly enriched in glioma tumor cells compared with other non-neuronal cells (Figures 2G–I).

Furthermore, following the four glioblastoma subtypes proposed by Neftel et al. (2019), scRNA-seq clustering was performed, primarily including four cell clusters: astrocyte (AC)-like, neural progenitor cell (NPC)-like, oligodendrocyte progenitor cell (OPC)-like, and mesenchymal (MES)-like. The TCGA-CL subtype aligns with the AC-like cell state. Our research findings suggested that *GJC1* is predominantly expressed in AC-like cells, showing a significant distinction from the other three cell subtypes (Figures 2J–L). This observation is consistent with the expression profile observed in bulk tumor sequencing, where *GJC1* is primarily upregulated in TCGA-CL and TCGA-ME subtypes. Therefore, our results indicated that *GJC1* could serve as a potential biomarker for GBM subtypes.

### 3.3 *GJC1* serves as an independent prognostic factor for the OS of patients with glioma

**Figure 3:**
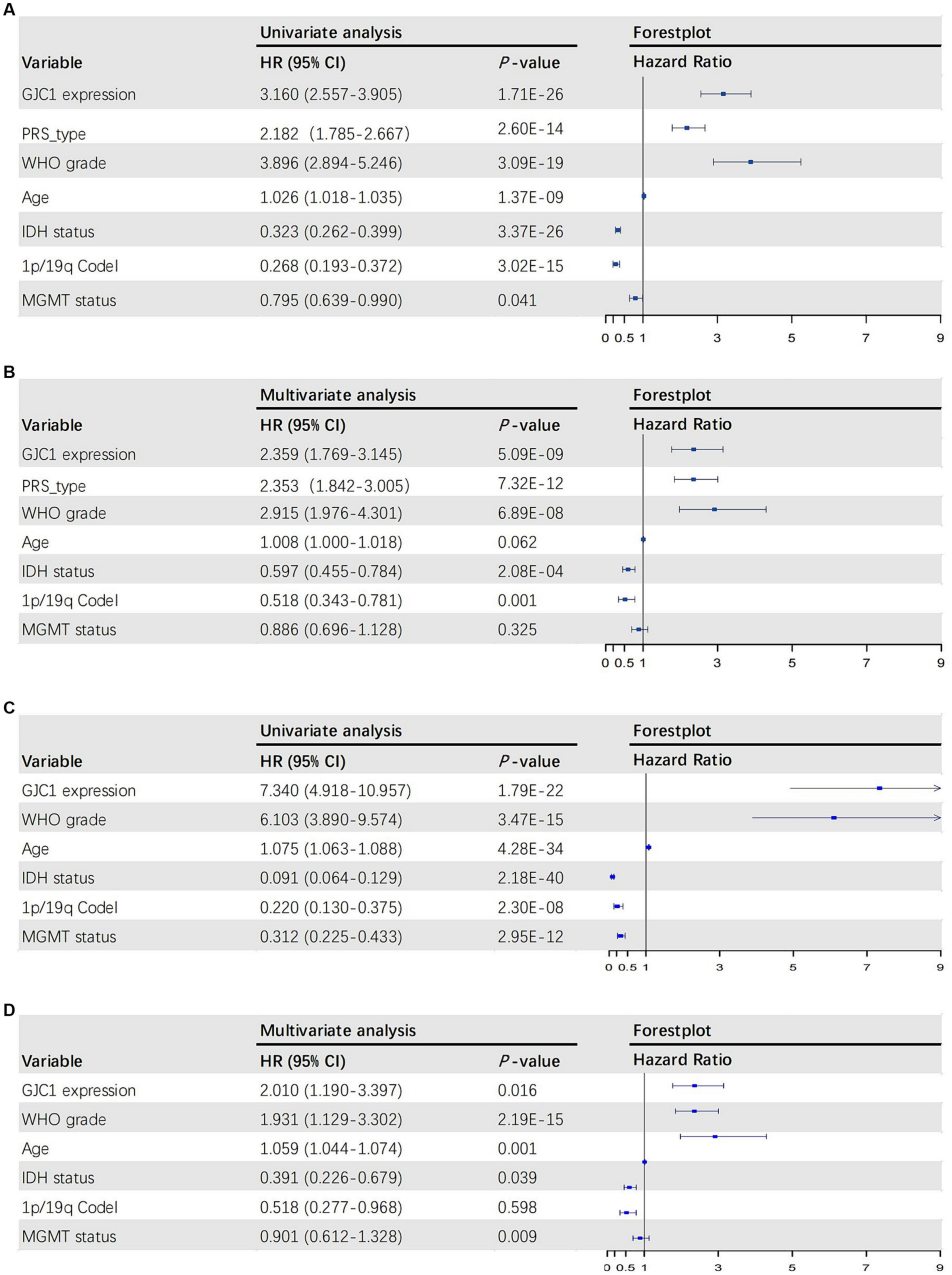
The univariate and multivariate Cox regression analyses in the CGGA and TCGA databases. **(A)** Forest plot of univariate analysis of prognostic parameters for OS in the CGGA database. **(B)** Forest plot of multivariate analysis of prognostic parameters for OS in the CGGA database. **(C)** Forest plot of univariate analysis of prognostic parameters for OS in the TCGA database. **(D)** Forest plot of multivariate analysis of prognostic parameters for OS in the TCGA database. OS, overall survival; CGGA, Chinese Glioma Genome Atlas; TCGA, The Cancer Genome Atlas; PRS, primary-recurrent status; WHO, World Health Organization; IDH, isocitrate dehydrogenase.

To further elucidate the prognostic value of *GJC1*, we performed both univariate and multivariate Cox regression analyses in the CGGA and TCGA databases. In the CGGA database, the multivariate Cox regression HR for *GJC1* expression was 2.359 (95% CI: 1.769–3.145). In the TCGA database, the multivariate Cox regression HR for GJC1 expression was 2.010 (95% CI: 1.190–3.397). In Cox regression analysis, *GJC1* expression emerged as an independent prognostic factor, distinct from known prognostic factors, such as WHO grading, age at diagnosis, *IDH* mutation status, 1p/19q co-deletion status, and *MGMT* promoter methylation status (Figures 3A–D).

### 3.4 *GJC1* may impact glioma progression by modulating the cell-cycle signaling pathway

**Figure 4:**
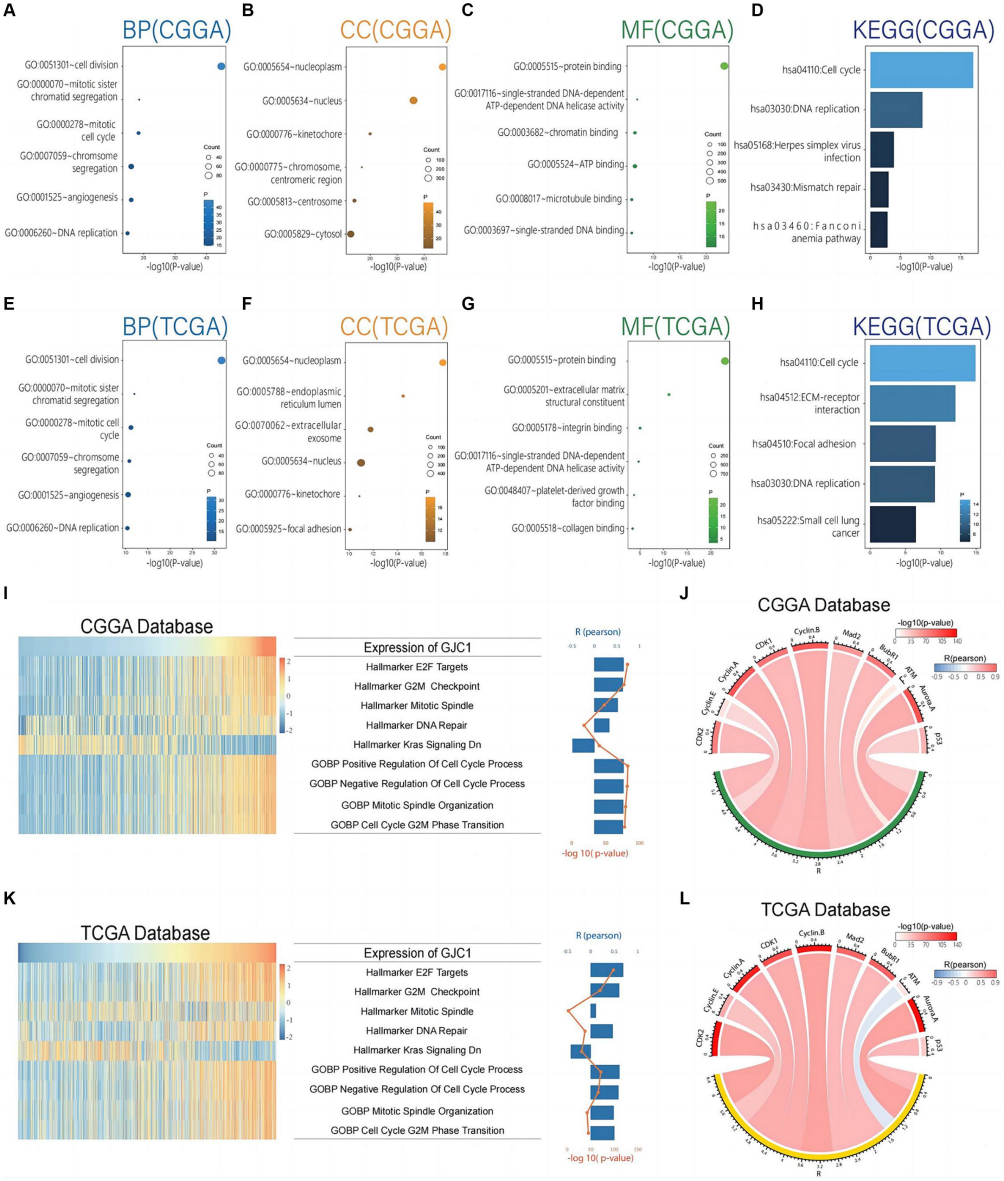
*GJC1* is closely linked to cell-cycle regulation in gliomas. **(A–C)** Biological process (BP), cellular component (CC), and molecular function (MF) GO terms predominantly associated with *GJC1*-correlated genes in the CGGA database. **(D)** KEGG pathway analysis of *GJC1*-correlated genes in the CGGA database. **(E–G)** BP, CC, and MF GO terms predominantly associated with *GJC1*-correlated genes in the TCGA database. **(H)** KEGG pathway analysis of *GJC1*-correlated genes in the TCGA database. **(I,K)** Correlation analysis between *GJC1* expression and enrichment scores of cell cycle regulation-related gene sets. The heatmap illustrates *GJC1* expression and the enrichment scores of cell cycle regulation-related gene sets for each patient in the CGGA and TCGA databases. Samples are arranged in ascending order of *GJC1* expression. The column and line graphs on the right display the Pearson’s *R*-value and *p*-value. **(J,L)** Pearson correlation analysis between *GJC1* expression and key cell-cycle proteins in the CGGA and TCGA databases. The width of the band indicates the *R*-value, while the color represents the p-value.

To investigate the biological functions of *GJC1* in gliomas, we conducted Pearson correlation analysis on glioma transcriptome data from TCGA and CGGA databases to identify genes most strongly correlated with *GJC1* (|R| > 0.5, *p* < 0.05). Subsequently, we performed GO and KEGG analyses based on the curated gene sets. The biological processes most closely linked to *GJC1*-correlated genes encompassed critical cellular activities, including cell division, mitotic sister-chromatid segregation, chromosome segregation, and DNA repair (Figures 4A,E). Furthermore, the cellular components most strongly associated with *GJC1*-correlated genes were identified as the nucleoplasm and nucleus (Figures 4B,F). The molecular functions most closely associated with *GJC1*-correlated genes were protein binding and single-stranded DNA-dependent ATP-dependent DNA helicase activity (Figures 4C,G). The signaling pathways most related to *GJC1* were the cell cycle and DNA replication (Figures 4D,H). These findings strongly support an oncogenic role for *GJC1* in promoting glioma proliferation and growth by enhancing cell-cycle progression.

The GO and KEGG enrichment analyses provided functional insights into the role of *GJC1* in glioma pathogenesis. We further investigated the role of *GJC1* in gliomas using GSVA. GSVA revealed a strong correlation between *GJC1* expression and gene sets and pathways related to cell-cycle regulation and mitosis in glioma (Figure 4I). Specifically, we observed highly significant positive correlations between *GJC1* expression and gene sets representing E2F targets, G2/M checkpoint, mitotic spindle, DNA repair, positive regulation of cell cycle processes, negative regulation of cell cycle, mitotic spindle organization, and G2/M phase transition. The most significant correlations were observed with pathways involved in cell-cycle progression (Pearson *R*-value: 0.65; *p* < 0.001). Additionally, *GJC1* was negatively correlated with hallmark_kras_signaling_dn, which indicated a potential mechanism for suppressing KRAS signaling in glioma, possibly contributing to tumor suppression or influencing therapeutic response. The same analysis of the TCGA data yielded the same results, further confirming the robustness of the above conclusions (Figure 4K). To further investigate how GJC1 is involved in cell proliferation pathways and glioma subtypes, we conducted GSVA analysis on non-neuronal cell proliferation across the four Glioma Transcriptome Subtypes. The analysis indicated that GJC1 expression levels are correlated with the cell proliferation of Neuroblast, Neural Precursor Cell, CD4 Positive Alpha Beta T Cell, and Mesenchymal Stem Cell populations. This correlation is particularly significant in the TCGA-CL and TCGA-ME subtypes (Supplementary Figure S2).

Based on the protein correlation analysis data, we discovered significant correlations between *GJC1* expression and multiple key cell-cycle regulatory proteins. Specifically, *GJC1* expression levels were positively correlated with CDK2 (*r* = 0.780, *p* < 0.001), Cyclin E (*r* = 0.526, *p* < 0.001), Cyclin A (*r* = 0.760, *p* < 0.001), CDK1 (*r* = 0.701, *p* < 0.001), Cyclin B (*r* = 0.776, *p* < 0.001), Mad2 (*r* = 0.665, *p* < 0.001), and BubR1 (*r* = 0.674, *p* < 0.001) (Figure 4J). Similar correlations were observed in the TCGA database (Figure 4I). These findings suggested that *GJC1* may play a crucial role in governing cell-cycle progression in glioma by interacting with these critical regulators. Moreover, we also observed a negative correlation between *GJC1* and ATM in the TCGA database, a key protein involved in DNA damage response and repair, with a Pearson correlation coefficient of -0.338 and a *p* < 0.001 (Figure 4I). Such negative correlations did not appear in the analysis of the CGGA database; however, this suggested that *GJC1* might also be involved in the regulation of DNA damage pathways in glioma. Our findings provided new insights into the intricate interactions between *GJC1* and glioma cell-cycle mechanisms and DNA damage response and offer the possibility of further investigating the potential of *GJC1* as a therapeutic target for glioma.

### 3.5 Drug sensitivity analysis of *GJC1*

**Figure 5:**
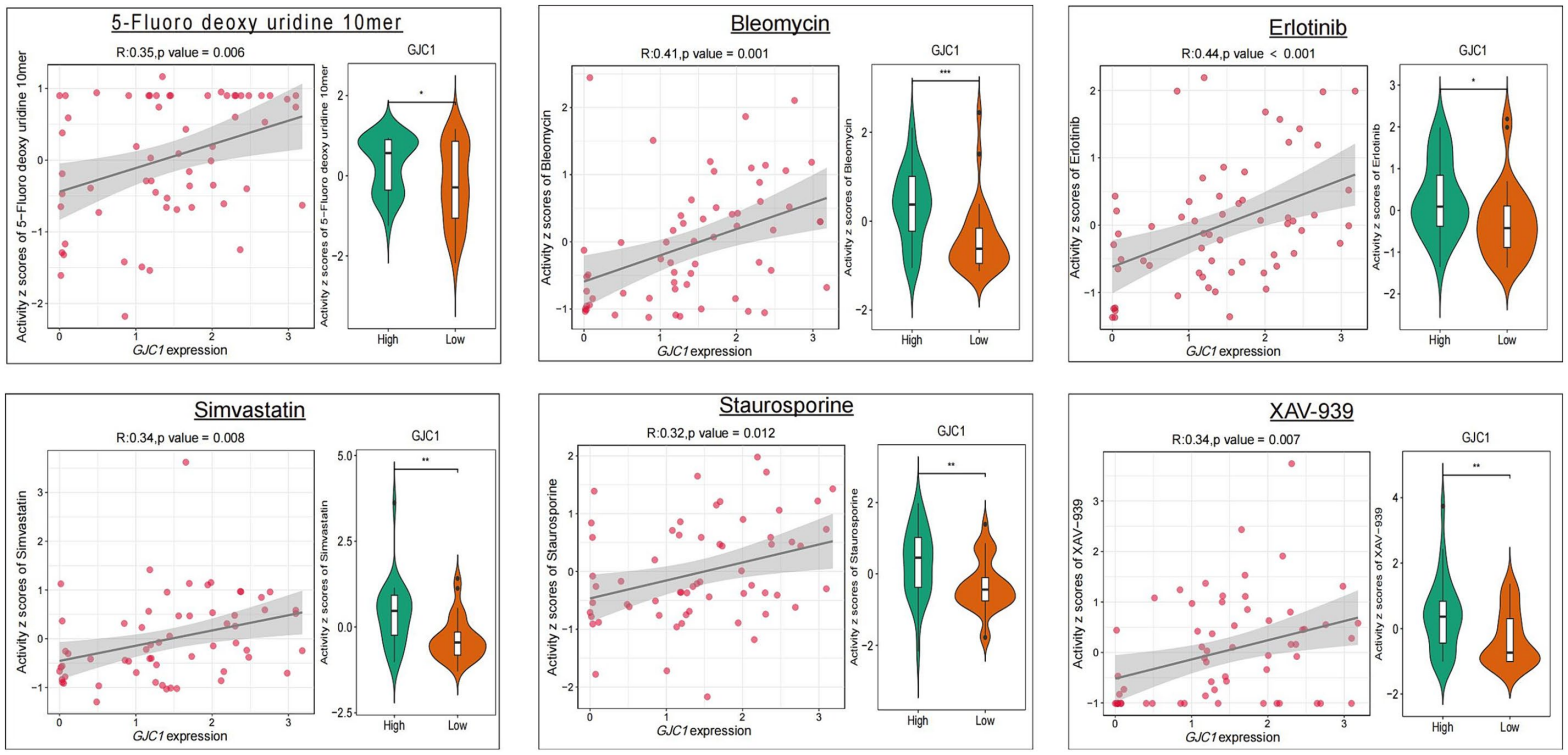
The relationship between *GJC1* expression and the top nine anticipated medication responses. The relationship between drug IC_50_ values and *GJC1* expression is listed on the left. Gene expression data were stratified into high and low groups according to the median gene expression. Differential drug efficacy assays for the *GJC1* high-and low-expression subgroups are presented in violin plots on the right.

CellMiner was utilized to explore the correlation between *GJC1* and drug response (Figure 5). *GJC1* expression demonstrated an inverse correlation with drug responsiveness among patients treated with 5-fluorodeoxyuridine, bleomycin, erlotinib, fludarabine, ibrutinib, sapitinib, simvastatin, staurosporine, and XAV-939. High and low expression groups were categorized based on median *GJC1* expression in the CellMiner database. These findings suggested a potential link between *GJC1* expression and drug resistance in tumor cells. These results provided valuable insights for the treatment of patients with glioma experiencing high *GJC1* expression.

## 4 Discussion

We identified *GJC1* as a groundbreaking oncogenic factor in glioma. Our investigation revealed significant overexpression of *GJC1* in glioma, which demonstrated a compelling correlation with disease progression. Clinically, elevated *GJC1* expression emerged as an independent indicator of a poor prognosis for individuals with glioma, offering a valuable avenue for its utilization as a prognostic biomarker to predict patient OS.

GJ intercellular communication is recognized for its pivotal role in maintaining tissue homeostasis and orchestrating the development and differentiation of various tissues (Totland et al., 2020). Cx43 has been extensively investigated, and connexins are broadly distributed within the nervous system. Cx43 has been associated with cancer recurrence, metastatic spread, and reduced survival rates. Its counterpart, Cx45, is also found in the central nervous system, particularly during periods of growth and development. Only one study has suggested a link between abnormalities in Cx45 and the development of familial atrial fibrillation; however, our curiosity led us to explore the potential role of *GJC1*, the gene responsible for encoding Cx45, in tumors, with a particular focus on gliomas, the most common type of central nervous system tumor. This study presents evidence demonstrating that abnormal overexpression of *GJC1* independently contributes to a poorer prognosis for gliomas. *GJC1* holds promise as a valuable marker for identifying gliomas linked with a less favorable prognosis. Furthermore, through scRNA-seq data analysis, we identified a significant upregulation of *GJC1* expression primarily within glioma tumor cells. *GJC1* is strongly correlated with cell cycle-related proteins, and its most related signaling pathways are cell-cycle regulation and DNA replication.

Cell-cycle dysregulation is a hallmark of gliomas (Qin et al., 2022). Glioma cells often exhibit dysregulated proliferation, leading to uncontrolled growth. Glioma cells exhibit abnormal regulation of the cell cycle, including changes in CDKs and cyclins, which are essential for cell-cycle progression. *GJC1* identification as a prognostic marker for glioma, particularly its enrichment in the cell-cycle pathway as revealed through KEGG and GO analyses, provides valuable insights into the molecular mechanisms underlying glioma progression. The strong correlation between *GJC1* and key cell-cycle proteins underscores its pivotal role in the dysregulated cell cycle observed in glioma. These findings open doors for the development of targeted therapies that may involve regulating GJC1 to restore normal cell-cycle control in glioma cells, potentially inhibiting their uncontrolled proliferation.

Furthermore, we observed a positive correlation between *GJC1* expression and sensitivity to several anti-cancer drugs. Our analysis revealed that, alongside classical chemotherapeutic and targeted agents, simvastatin and staurosporine emerged as prominent among the top nine relevant drugs. Previous reports have documented the anti-tumor potential of these drugs. The anti-tumor mechanism of action of these two drugs is related to cell-cycle regulation. Simvastatin, alongside other statins, classically functions by inhibiting HMG-CoA reductase during the initial steps of the mevalonate pathway.

Consequently, simvastatin is a frequently employed pharmacological agent for lipid-lowering purposes in clinical settings (LIPID Study Group, 1998). In a colon cancer study, simvastatin was shown to mediate tumor-cell apoptosis by downregulating CDK4, which is a key protein in cell-cycle regulation (Gajjar et al., 2023). Staurosporine exhibits biological activities spanning from antifungal to antihypertensive (Rüegg and Burgess, 1989). At lower concentrations, staurosporine induces specific cell-cycle effects, impeding cells from progressing through the G1 or G2 phase of the cell cycle, depending on the cell type (Lin et al., 1992).

However, our study has some limitations that need to be addressed in future research. Our findings are based on bioinformatic analyses of public databases, which may not reflect the actual biological situation of patients with glioma. Therefore, experimental validation of *GJC1* expression and function in glioma samples and cell lines is necessary to confirm our results. Additionally, we did not investigate the molecular mechanisms by which *GJC1* regulates the cell-cycle pathway and its key proteins. Further studies are needed to elucidate how *GJC1* affects the cell cycle at the molecular level and whether specific drugs or interventions can target it.

In summary, we systematically investigated the influence of clinicopathological features, molecular subclasses, and prognosis of gliomas on *GJC1* expression patterns. We analyzed the biological processes and markers associated with *GJC1* in tumor cells and further performed drug correlation analysis. Moreover, all the specific mechanisms of drug action obtained from the drug correlation analysis were related to the cell cycle, further supporting the influence of *GJC1* on cell-cycle regulation. These results initially revealed the critical role of *GJC1* in the cell cycle. Further studies are warranted to investigate *GJC1* as a novel biomarker or therapeutic mediator in gliomas or other tumor types. Research targeting *GJC1* holds significant potential.

## Data availability statement

The original contributions presented in the study are included in the article/Supplementary material, further inquiries can be directed to the corresponding author/s.

## Author contributions

XJ: Formal analysis, Software, Writing – original draft, Writing – review & editing, Investigation, Methodology. XC: Funding acquisition, Writing – original draft, Conceptualization, Data curation. GL: Writing – review & editing, Investigation, Visualization. KM: Writing – review & editing, Resources, Supervision. JHY: Software, Writing – original draft, Validation. XZ: Funding acquisition, Visualization, Writing – original draft. SC: Writing – review & editing, Conceptualization. JNY: Funding acquisition, Supervision, Writing – review & editing, Conceptualization, Resources.
