# Supplementary material for: Discovery of GJC1 as a prognostic biomarker in glioma cells: insights into its cell-cycle relationship and differential expression in non-neuronal cells
Source: Front Cell Neurosci. 2024 Sep 18;18:1440409. doi: 10.3389/fncel.2024.1440409 (PMC11445671; doi:10.3389/fncel.2024.1440409)
Supplement: Supplementary file 1 [file Data_Sheet_1.DOCX]

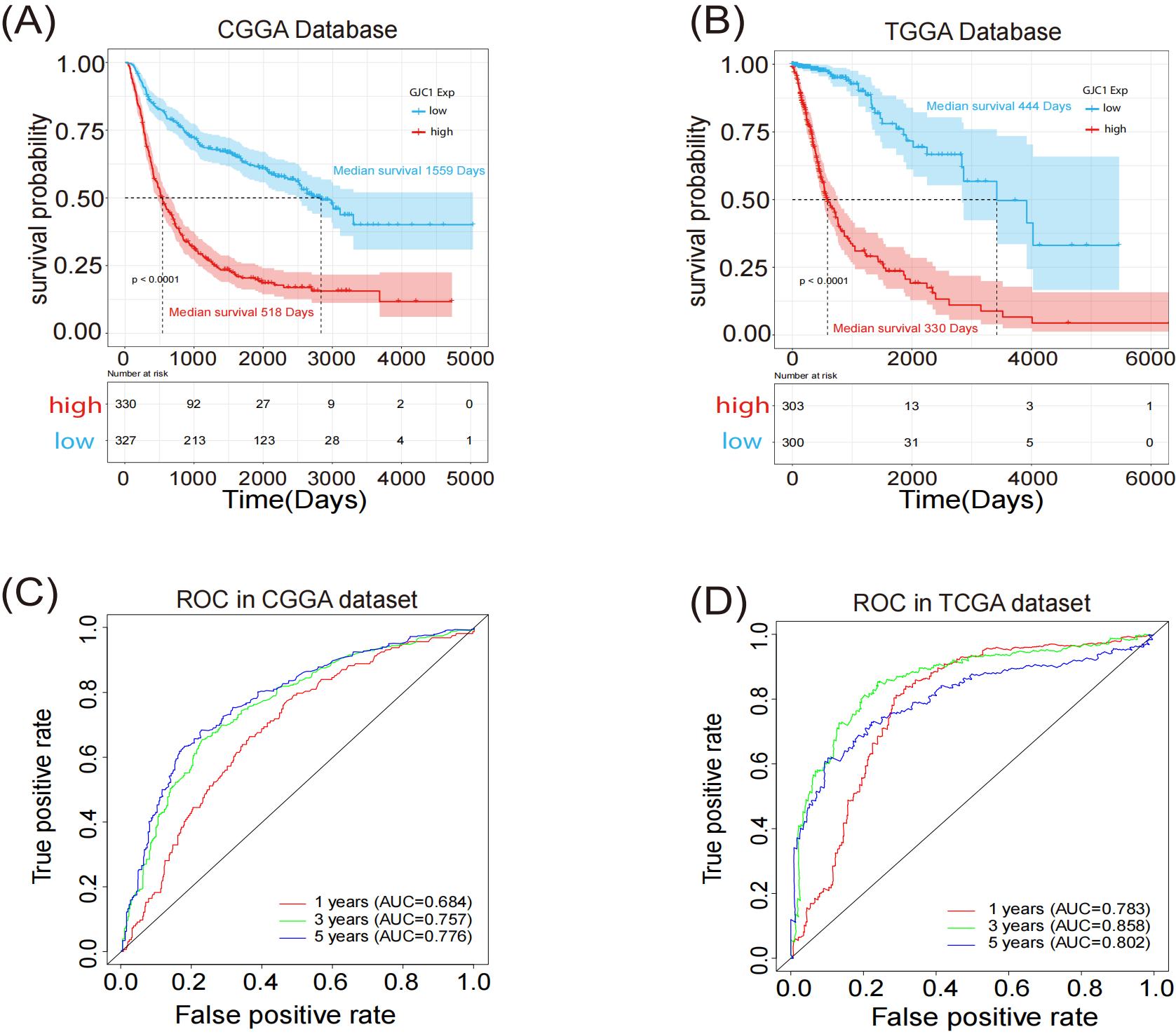


**Supplementary Figure 1.** The prognostic and diagnostic potential of *GJC1* in glioma. (A, B) Survival analysis of *GJC1* in gliomas using the Kaplan–Meier method for OS. (C, D) The significance of the prognostic value was evaluated using a log-rank test. Time-dependent receiver operating characteristic (ROC) curves were generated for survival rates over 1, 3, and 5 years. OS, overall survival.
