# Supplementary material for: Discovery of GJC1 as a prognostic biomarker in glioma cells: insights into its cell-cycle relationship and differential expression in non-neuronal cells
Source: Front Cell Neurosci. 2024 Sep 18;18:1440409. doi: 10.3389/fncel.2024.1440409 (PMC11445671; doi:10.3389/fncel.2024.1440409)
Supplement: Supplementary file 2 [file Data_Sheet_2.DOCX]

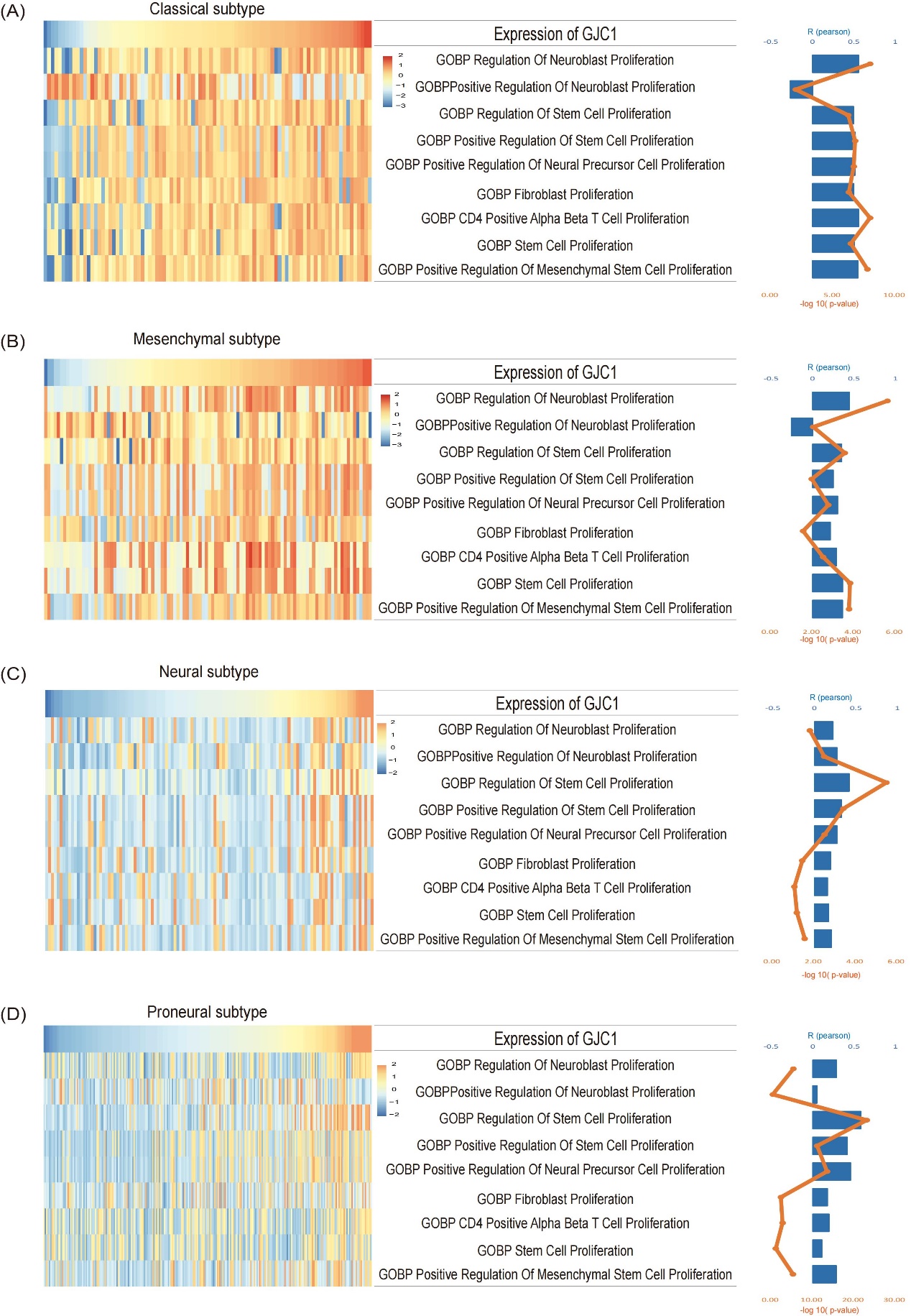


**Supplementary Figure 2.** Correlation analysis between *GJC1* expression and enrichment scores of non-neuronal cells proliferation-related gene sets. The heatmap illustrates *GJC1* expression and the enrichment scores of non-neuronal cells proliferation-related gene sets for each patient in the four glioma transcriptome Subtypes. Samples are arranged in ascending order of *GJC1* expression. The column and line graphs on the right display the Pearson’s R-value and P-value.
